# Imageless Robotic Arm-Assisted Total Knee Arthroplasty: Workflow Optimization, Operative Times, and Learning Curve

**DOI:** 10.7759/cureus.78880

**Published:** 2025-02-11

**Authors:** Sanjay B Londhe, Kunal Patel, Govindkumar Baranwal

**Affiliations:** 1 Department of Orthopaedics, Criticare Asia Hospital, Mumbai, IND

**Keywords:** imageless systems, learning curve, robotic-assisted total knee arthroplasty, soft-tissue release, surgical time, velys robotic-assisted system

## Abstract

Background

Robotic arm-assisted total knee arthroplasty (RATKA) offers several advantages, including precise restoration of mechanical or kinematic alignment, accurate bone resections, reliable implant size prediction, alignment optimization, and dynamic gap balancing. However, a key concern among arthroplasty surgeons is the perceived increase in operative time associated with adopting this technology. This study describes the step-by-step surgical workflow of imageless RATKA and evaluates the surgical times and learning curve associated with this technique.

Methods

This study is a retrospective analysis of the data of the first 60 cases of imageless RATKA done between February 2023 and November 2024 at a single surgical center by the same surgical team. Patients undergoing imageless RATKA for Kellgren and Lawrence grade 4 osteoarthritis were included, while those with prior knee surgery or high tibial osteotomy were excluded. All procedures utilized the DePuy Attune implant with a tibia-first surgical workflow, performed via a midline vertical incision and medial parapatellar arthrotomy. Surgical times were recorded and analyzed by an independent observer not involved in the surgeries. The 60 cases were divided into four groups of 15 cases (group 1 consisted of the first 15 cases, i.e., case number 1 to case number 15; group 2 consisted of the next consecutive 15 cases, i.e., case number 16 to case number 30; group 3 consisted of case number 31 to case number 45; and group 4 consisted of the last 15 cases, i.e., case number 46 to case number 60) each to evaluate the learning curve and calculate mean surgical times.

Results

The surgical times (in minutes) of the various groups were as follows: group 1 (0-15 cases) = 96.27 ± 4.46; group 2 (16-30 cases) = 91.07 ± 3.75; group 3 (31-45 cases) = 88.67 ± 3.58; group 4 (46-60 cases) = 86.13 ± 3.66. Comparison of means shows p values of 0.005, 0.03, and 0.09 between group 1 and 2, group 2 and 3, and group 3 and 4, respectively, indicating normalization of the operative time and a learning curve of 15 cases.

Conclusion

By following a standardized and reproducible tibia-first workflow, the operative time for imageless RATKA normalizes roughly after 15cases, i.e., group 2 onwards. This suggests that surgical time should not be a barrier for surgeons considering the adoption of this technology. The findings support the feasibility and efficiency of integrating robotic-assisted systems into routine arthroplasty practice.

## Introduction

The adoption of robotic arm-assisted (RA) technologies in total knee arthroplasty (TKA) has been steadily increasing, driven by the need for improved precision, reproducibility, and clinical outcomes in knee replacement surgeries [1]. For instance, between 2015 and 2020, there was a substantial (601.2%) increase in RATKAs, highlighting the rapid integration of this technology into surgical practice [2]. In the United States, the prevalence of RATKA procedures has grown notably. According to the American Joint Replacement Registry, robotic systems accounted for 13% of all TKAs as of 2022, reflecting a significant uptick in utilization [3]. Projections indicate that this upward trend will continue. A study analyzing data from 112,161 TKA procedures found that 7.2% were technology-assisted. The same study forecasts that by 2032, the proportion of technology-assisted TKAs could reach 50%, suggesting a substantial shift toward the adoption of advanced technologies in knee arthroplasty [4]. This trend gained significant momentum after Stryker (Stryker Orthopaedics, Kalamazoo, MI, USA) introduced the Mako robotic arm system for TKA on March 14, 2017, at the American Academy of Orthopaedic Surgeons (AAOS) annual meeting in San Diego, California. This launch marked the Mako System as the first robotic technology capable of performing total knee, total hip, and partial knee replacements [5,6]. Initially designed for unicompartmental knee arthroplasty, the Mako robotic arm demonstrated substantial advantages in terms of precision, alignment, and soft tissue preservation, which led to its adaptation for RATKA [5,6]. Since then, other major manufacturers have developed their own RA systems, further expanding the options available for surgeons [7]. 

Robotic-assisted technologies can be broadly categorized into two types: image-based systems, which require preoperative imaging such as computed tomography (CT) or magnetic resonance imaging (MRI), and imageless systems, which rely on intraoperative data capture and do not require additional imaging. Prominent image-based systems include Mako (Stryker Orthopaedics, USA), Cuvis Joint (Curexo, Republic of Korea), and MISSO (Meril Healthcare Pvt. Ltd., India). Imageless systems, such as Navio and Cori (Smith & Nephew, USA), ROSA (Zimmer Biomet, USA), and Velys (Johnson & Johnson Medtech, USA), enable real-time mapping of the knee anatomy during surgery without the need for preoperative imaging. 

The reported benefits of RATKA include enhanced precision in restoring the mechanical or kinematic axis of the lower limb, improved implant sizing, and reduced need for extensive soft tissue release [8]. A recent retrospective review of 100 cases demonstrated that robotic assistance led to significantly reduced soft tissue releases, optimized implant positioning, and more consistent gap balancing compared to conventional techniques, supporting the reproducibility and precision of this approach [8]. These advantages contribute to better alignment, optimal soft tissue balance, and reduced variability in surgical outcomes. Studies have shown that RTKA offers improvements in short-term clinical outcomes compared to conventional TKA, such as faster recovery, reduced post-operative pain, and higher patient satisfaction [9-15]. Despite these advantages, apprehensions among experienced arthroplasty surgeons persist regarding the potential for increased operative time and decreased operating room efficiency [13,16]. 

In this context, the Velys Robotic-Assisted System (VRAS) developed by DePuy Synthes introduces a new approach to RTKA. The VRAS is an imageless system that eliminates the need for preoperative CT or MRI imaging, relying instead on intraoperative data to capture both the bony anatomy and the soft tissue envelope of the knee. This enables surgeons to achieve accurate implant placement, alignment, and medial and lateral gap balancing throughout the knee’s range of motion (from full extension to 30°, 60°, 90°, and 120° of flexion). The VRAS offers a surgeon-driven workflow, allowing real-time validation of limb alignment and gap balancing intraoperatively. 

In this study, we provide an overview of VRAS technology, with a specific focus on its intraoperative workflow optimization. We also analyze the surgical times and learning curve associated with the first 60 cases performed by a single surgical team. By evaluating these parameters, we aim to provide insights into the practical implications and efficiency of integrating this novel imageless robotic system into routine arthroplasty practice.

## Materials and methods

This study is a retrospective analysis of the data of the first 60 cases of imageless RATKA done between February 2023 and November 2024 performed at Criticare Asia Multi-Speciality Hospital, a surgical center in Maharashtra, India, by the same surgical team. The inclusion criteria were patients undergoing imageless RTKA for Kellgren and Lawrence grade 4 osteoarthritis. Patients with a history of prior knee surgery or high tibial osteotomy were excluded. The tibial resection was performed first following the tibia-first workflow.

Surgical times for all 60 cases were recorded and analyzed by an independent observer who was not involved in the surgical procedures. To evaluate operative time trends, the 60 cases were divided into four groups of 15 cases each (group 1 consisted of the first fifteen cases i.e. case number 1 to case number 15, group 2 consisted of the next 15 consecutive cases i.e. case number 16 to case number 30, group 3 consisted of case number 31 to case number 45 and group 4 consisted of the last 15 cases i.e. case number 46 to case number 60). The mean operative time and standard deviation were calculated for each group. Statistical comparisons of mean operative times between groups were conducted using Student’s t-test, with a P-value <0.05 considered statistically significant.

Surgical technique

We hereby describe the patient-specific RATKA technique with the VRAS system.

This approach incorporates per-operative capture of the bony anatomy and soft tissue envelope of the arthritic knee to achieve functional anatomical restoration. The primary objective is to ensure well-balanced medial and lateral gaps throughout the knee's range of motion (ROM) at full extension and flexion angles of 30°, 60°, 90°, and 120°. By minimizing soft tissue releases, this technique aims to maintain the natural integrity of the knee’s soft tissue envelope.

All procedures were performed using the Attune implant system (DePuy Synthes, Johnson & Johnson Medtech, USA). A tourniquet was used in all patients. The same surgical team carried out all cases using a medial parapatellar arthrotomy approach. After arthrotomy, the anterior cruciate ligament (ACL), posterior cruciate ligament (PCL), medial and lateral menisci, and osteophytes from the femur and tibia were excised.

Tracker Pin Placement and Registration

Tracker pins were inserted into both the femur and tibia, with two pins placed in each bone. Pins were 4 mm in diameter and available in three lengths: 100 mm, 125 mm, and 175 mm. For most patients, 125 mm pins were used; however, for obese or overweight individuals, 175 mm pins were preferred to prevent soft tissue impingement and ensure unobstructed visualization of the arrays and camera. Pins were inserted within the surgical incision anteromedially at a 45° angle to minimize the risk of stress risers. Femoral and tibial tower trackers were attached to these pins.

**Figure 1:**
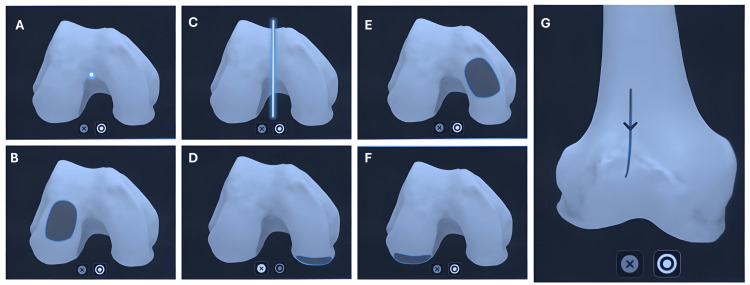
Anatomical landmarks and regions on the femur used for registration in the Velys robotic-assisted procedure. Panel A: femur center; Panel B: femur distal lateral condyle; Panel C: Whitside’s line; Panel D: femur posterior medial condyle; Panel E: femur distal medial condyle; Panel F: posterior lateral condyle; Panel G: anterior femur cortex registration.

Bony landmarks, including the hip, knee, and ankle (HKA) centers, were registered, and a 3D mapping of the distal femur and proximal tibia was performed using a pointer probe. The following registration points were captured on the femur: femoral center, Whiteside’s line, distal medial condyle, distal lateral condyle, posterior medial condyle, posterior lateral condyle, and anterior femoral cortex (Figure 1).

**Figure 2:**
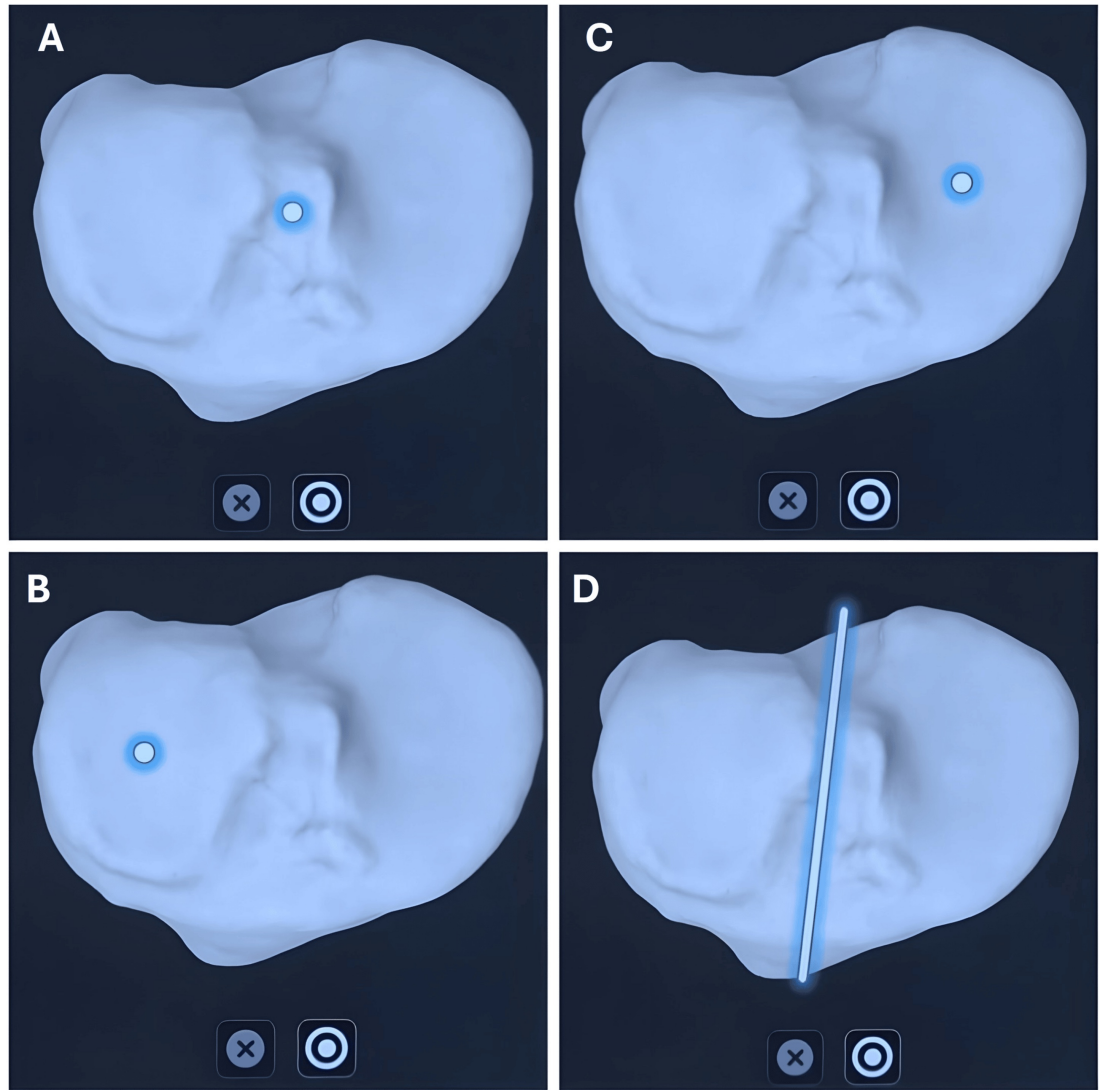
Anatomical landmarks and regions on the tibia used for registration in the Velys robotic-assisted procedure. Panel A: tibia center; Panel B: medial tibial plateau; Panel C: lateral tibial plateau; Panel D: line parallel to the upper surface of the tibia.

For the tibia, the tibial center, medial tibial plateau, lateral tibial plateau, and line parallel to the tibial upper surface (Figure 2) were captured.

**Figure 3:**
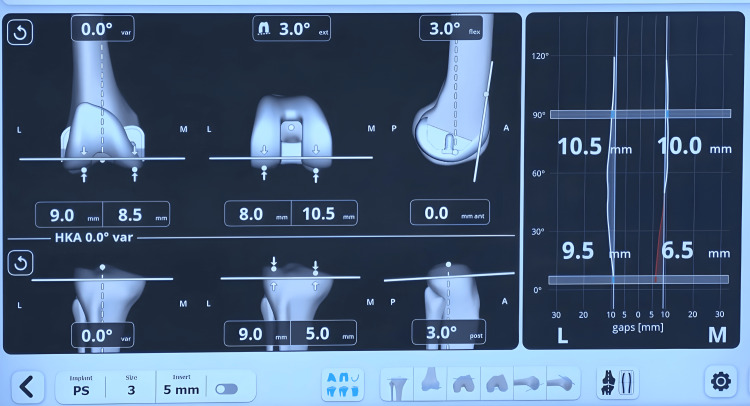
Manufacturer’s software showing the femur implant size and default femur component flexion and external rotation

Upon registration of these points, the robotic system software suggests the likely femur implant size (manufacturer’s plan) (Figure 3).

The distal femoral resection was planned perpendicular to the mechanical axis, at the appropriate level to fit the implant, ensuring posterior offset restoration without anterior cortex notching. The default plan sets the femoral component in 3° of flexion and 3° of external rotation (Figure 3).

The tibial resection was performed first as this approach allows for more physiologically optimized gap adjustments by manipulating the femur in multiple planes. The upper tibial resection is carried out by removing 9 mm from the unaffected tibial condyle. The upper tibia cut is usually set at 0° of varus/ valgus. In patients with severe preoperative varus deformity (>20°), we prefer to cut the proximal tibia in 2˚-3° of varus to achieve functional alignment of the limb. This in turn results in minimized soft tissue releases.

Soft Tissue Balancing and Gap Assessment

**Figure 4:**
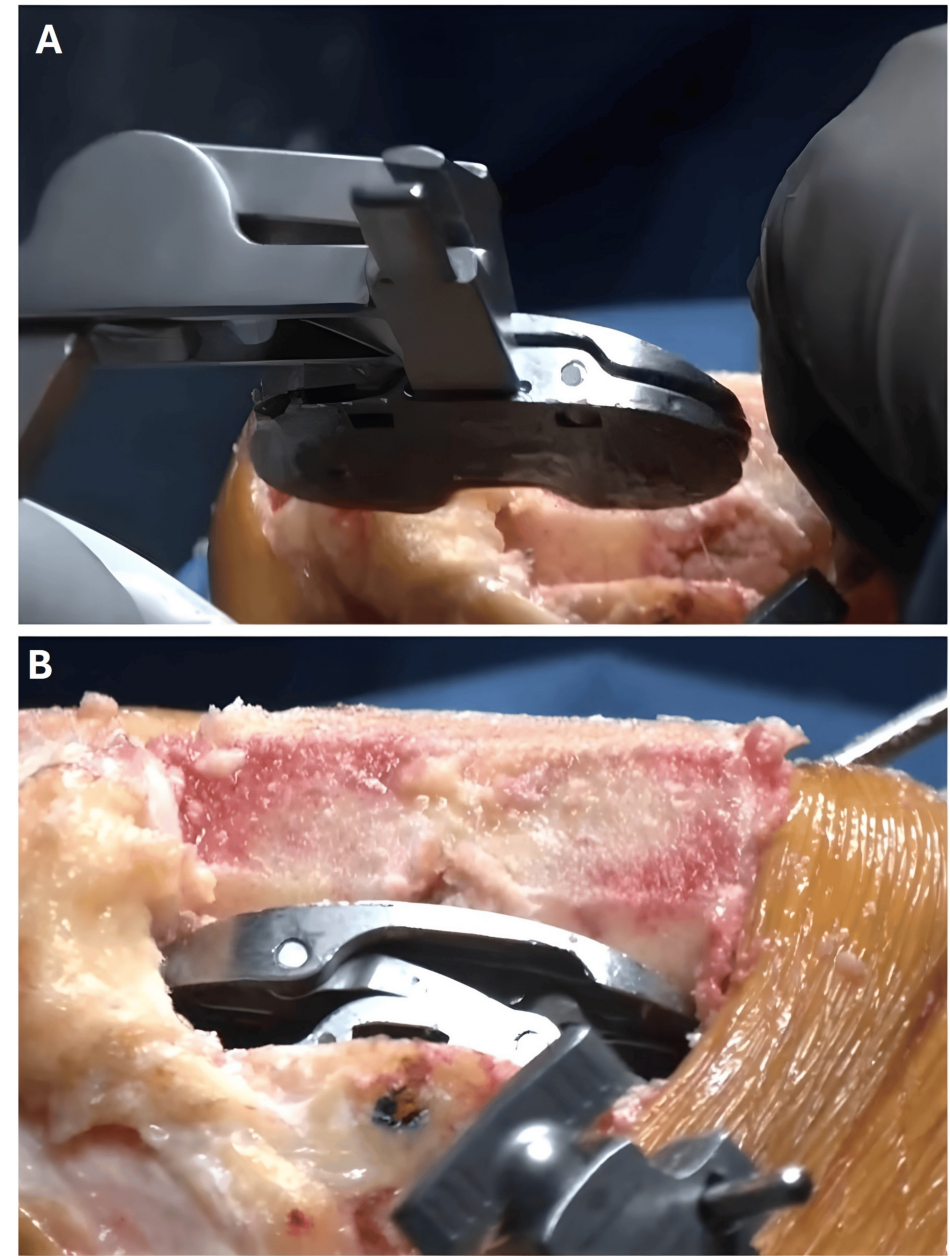
The sensor tensor device used during the Velys robotic-assisted procedure to assess and optimize soft tissue balance. Panel A: the sensor tensor instrument being inserted with the help of an introducer; Panel B: sensor tensor instrument being introduced between the femur and tibia in flexion.

**Figure 5:**
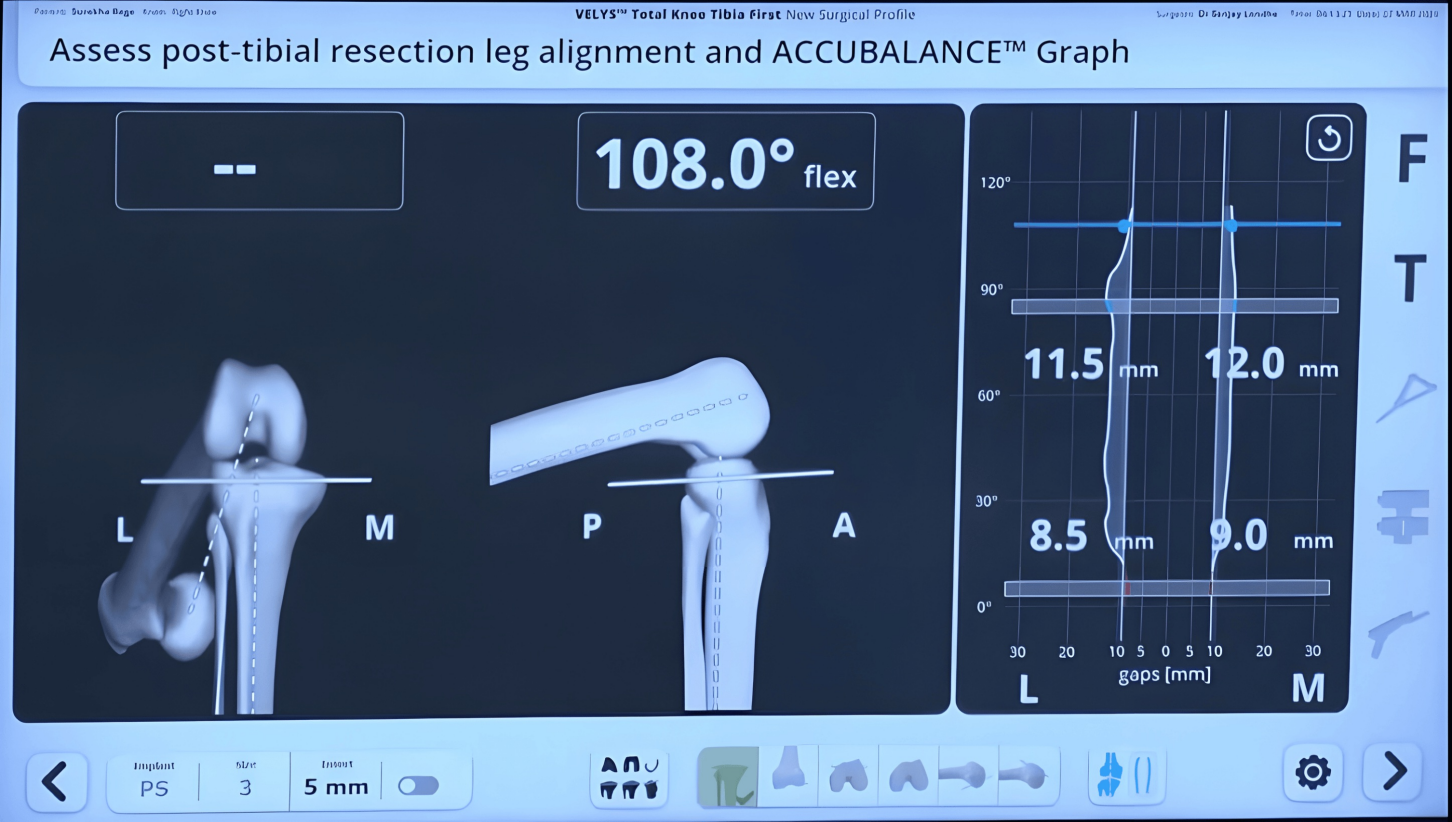
Pre-adjustment captured gaps in 0°-30°-60°-90° of the flexion

A sensor tensor device was placed between the resected tibial surface and the femur to measure medial and lateral gaps throughout the ROM, including full extension and flexion at 30°, 60°, 90°, and 120° (Figures 4, 5).

**Figure 6:**
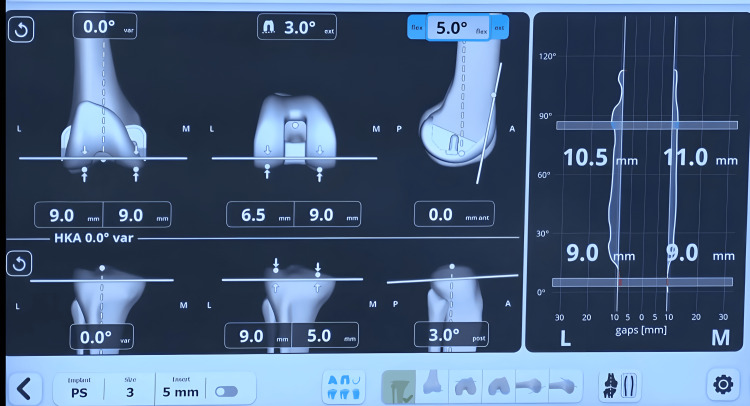
The captured joint gaps from full extension to full flexion following the final adjustments post robotic-assisted total knee arthroplasty. The visual representation emphasizes the achieved balance and alignment across the knee's range of motion.

The surgeon can fine-tune several femoral variables, including adjusting the distal femur cut, setting the distal femur in 1° to 3° of varus or valgus, internally or externally rotating the femoral implant, modifying the degree of flexion, and shifting the femoral component anteriorly or posteriorly to achieve optimal alignment and balance (Figure 6).

The goal was to achieve equal or near-equal medial and lateral gaps (±1 mm) in full extension and slight lateral laxity of 1-1.5 mm at 90° flexion.

Surgical Boundaries

Specific outer limits have been established for femoral and tibial resections to ensure optimal alignment and outcomes. The upper tibia can be resected with a maximum of 3° of varus, particularly in cases of severe preoperative varus deformities, and the tibial slope can be increased up to 5°. For the distal femur, the maximum resection is limited to 3° of varus or valgus, with the combined varus angle of the distal femur and proximal tibia not exceeding 5°. The femoral component allows for up to 7° external rotation provided anterior cortex notching is avoided, while internal rotation is capped at 3°, primarily for valgus knees. In addition, the femoral component can be positioned with a maximum of 6° flexion.

Final Execution and Component Implantation

Once the surgical plan was finalized, bone cuts were performed using a handheld robotic-assisted saw. The accuracy of cuts was verified with the pointer probe. Flexion and extension gaps were re-evaluated using trial implants. If required, titrated soft tissue releases were performed to achieve the desired balance. Upon ensuring optimal gap balance, the final femoral and tibial components were implanted using standard surgical techniques.

## Results

The surgical time demonstrated a progressive decrease across groups with increasing surgeon experience. In group 1 (0-15 cases), the mean surgical time was 96.27 ± 4.46 minutes. This significantly decreased to 91.07 ± 3.75 minutes in group 2 (16-30 cases), with a P value of 0.005, indicating a statistically significant improvement in efficiency.

Further reductions were observed in group 3 (31-45 cases), where the surgical time was 88.67 ± 3.58 minutes (p = 0.03 compared to group 2). Although the mean surgical time in group 4 (46-60 cases) further decreased to 86.13 ± 3.66 minutes, the difference was not statistically significant when compared to group 3 (p = 0.09).

These findings suggest that surgical time improves as surgeons gain experience with the Velys robotic-assisted system, with a plateau in efficiency gains observed after 15 cases (Table 1).

**Table 1:** Comparison of the mean surgical times across four groups categorized by surgeon experience using the Velys robotic-assisted system.

Groups	Surgical time (mean ± SD), minutes	P value (student’s t-test)
Group 1 (0-15 cases)	96.27 ± 4.46	-
Group 2 (16-30 cases)	91.07 ± 3.75	0.005 (Between groups 1 and 2)
Group 3 (31-45 cases)	88.67 ± 3.58	0.03 (Between groups 2 and 3)
Group 4 (46-60 cases)	86.13 ± 3.66	0.09 (Between groups 3 and 4)

In addition, the operative time stabilized after the 15th case, indicating a learning curve of approximately 15 cases.

There were no complications related to femur or tibia tracker pins, intraoperative femoral notching, or iatrogenic fractures in any of the cases. In addition, none of the cases required abandoning the VRAS to convert to conventional TKA.

## Discussion

Robotic-assisted technology in TKA offers both advantages and challenges. The primary benefits include enhanced precision, improved implant sizing and alignment, and superior short-term clinical outcomes compared to conventional TKA [11]. However, drawbacks such as increased surgical time and a learning curve associated with adopting the new technique must also be considered [17]. The efficiency of the surgical workflow can be significantly improved by employing a trained, dedicated surgical team and adhering to well-defined procedural steps. Improvements are particularly evident as surgeons gain experience with tracker pin placement, registration of bony landmarks, and RA resection techniques.

The learning curve is a critical consideration when introducing any new surgical technique [18,19]. Several studies have highlighted that RATKA has a relatively short learning curve, particularly in terms of operative time. Huq et al. [11] analyzed 60 conventional TKAs and 60 RATKAs performed by a surgeon with prior cadaveric RATKA experience and observed a steep reduction in operative time after the seventh case, with no learning curve identified for implant accuracy or complication rates. Similarly, Plaskos et al. [12] reviewed 100 RATKAs and found that operative times were within five minutes of conventional TKA times after 20 cases. The grouping of cases in increments of 15 was based on the learning curve patterns observed in these studies on RATKA [11,12]. In our study, we observed a statistically significant reduction in surgical time after the first 15 cases and time between group 1 (0-15 cases) and group 2 (16-30 cases). Beyond this, no statistically significant difference was observed between group 2 and group 3 or between group 3 and group 4, suggesting that this number represents a key threshold in the learning curve. Our average surgical time, measured from incision to dressing placement, consistently remained under 91 minutes after the 15th case, reflecting improved efficiency and familiarity with the technique.

The VRAS has been recognized for its ability to achieve patient-specific outcomes in TKA by enabling precise intraoperative adjustments to implant alignment, positioning, and soft tissue balance through real-time data [20]. The study by Clatworthy N has emphasized the imageless workflow, which eliminates the need for preoperative CT or MRI imaging, along with the system’s efficiency and short learning curve, making it practical for routine clinical use. Our findings resonate with his observations, as we demonstrated that the Velys system facilitates a manageable learning curve, with surgical times stabilizing after approximately 15 cases, underscoring its efficiency in clinical practice. Moreover, our study adds a novel contribution by providing a detailed analysis of the stepwise intraoperative workflow and its role in optimizing surgical time. By quantifying operative time reductions with increased familiarity, we offer practical insights into how procedural experience with tracker placement, bony landmark registration, and intraoperative adjustments enhances efficiency, complementing Clatworthy’s focus on precision and patient-specific outcomes.

Recent literature further emphasizes the efficiency of RA systems like VRAS in optimizing surgical workflows. Lonner et al. have highlighted how streamlined robotic workflows improve surgical time and reduce learning curves [21], aligning with our findings. In addition, studies by Clatworthy et al. [20] show that VRAS provides significant advantages over burr-based robotic systems, such as faster bone resections and the ability to perform minor recuts without substantially increasing operative time. These capabilities ensure greater intraoperative adaptability while maintaining precision. Furthermore, the VRAS is associated with reduced hospital follow-ups with no early revisions [22].

While RATKA has yet to demonstrate clear superiority over conventional TKA in long-term functional outcomes [20,23], it offers several notable advantages. Multiple studies have reported improved implant positioning, better knee balance, and comparable or superior safety profiles in terms of avoiding iatrogenic soft tissue injuries [13,14]. Haddad et al. [15] demonstrated that RATKA is associated with reduced postoperative pain, lower analgesic requirements, and shorter hospital stays compared to conventional TKA. Kinematic alignment, as described by An et al. [24], preserves bone and soft tissue compared to mechanical alignment by aligning components to the patient’s native joint anatomy. While functional outcomes were not evaluated in this study, the ability of the VRAS to facilitate precise intraoperative adjustments aligns with the principles of kinematic alignment, potentially reducing the need for extensive soft tissue releases and complex adjustments. This bone- and tissue-preserving approach, combined with the system’s streamlined workflow, may contribute to reduced surgical times and a manageable learning curve, as observed in our cohort.

The VRAS offers unique advantages, such as the ability to make fine adjustments of 0.5-1 mm in resections and 1° in rotation or flexion/extension. Unlike burr-based robotic systems, the VRAS enables faster tibial and femoral resections and allows for minor recuts, such as increased depth or varus/valgus angle adjustments, without significant additions to operative time. The concept of constitutional varus, as introduced by Bellemans et al. [25], challenges the traditional notion that neutral mechanical alignment is ideal for all patients. Their findings suggest that a naturally occurring varus alignment is present in a significant portion of the population and that strict adherence to neutral alignment during TKA may disrupt natural biomechanics. These insights underscore the importance of individualized alignment strategies in TKA. Recent literature has highlighted the evolving role of RA systems in TKA, emphasizing their safety, precision, and adaptability to patient-specific alignment strategies. Londhe et al. demonstrated that with RATKA, one can reliably achieve accurate alignment and implant positioning with a favorable safety profile [26]. These findings align with our study, which underscores the efficiency and precision of the VRAS. The manageable learning curve observed in our study further supports the practical implementation of such advanced technologies in routine arthroplasty practice. In addition, Londhe et al. examined the efficacy of preoperative three-dimensional (3D) CT templating in predicting implant size and alignment in robotic-assisted TKA [27]. While our study utilized an imageless robotic system, the intraoperative workflow of VRAS achieved comparable precision in implant sizing and alignment without the need for preoperative imaging, simplifying the surgical preparation process. This emphasizes that imageless robotic systems can provide similar benefits to image-based systems while reducing logistical challenges and radiation exposure for patients. Furthermore, Clatworthy et al. [28] findings suggest that individualized alignment strategies, such as constitutional alignment, lead to improved joint balance and patient outcomes. The VRAS system in our study supports these individualized strategies by enabling fine-tuned adjustments to alignment and soft tissue balance intraoperatively, potentially reducing the need for extensive soft tissue releases. Hirschmann et al. [29] discussed the importance of transitioning from systematic alignment approaches to more individualized alignment strategies in TKA. Our study reinforces this perspective by demonstrating how the VRAS system facilitates personalized alignment adjustments during surgery. This capability aligns with the growing consensus that individualized approaches can achieve better functional outcomes and soft tissue balance, although long-term outcomes remain an area for further investigation.

This study provides valuable insights into the surgical workflow, learning curve, and efficiency of imageless RATKA using the VRAS, but certain limitations warrant consideration. The retrospective design, while based on prospectively collected data, may limit control over potential confounding factors; however, it allowed for a comprehensive evaluation of real-world clinical practices, enhancing the study's practical relevance. The single-center setting and consistent involvement of a single surgical team may limit generalizability but simultaneously strengthen the reliability and validity of the findings by ensuring uniformity in technique and workflow. Although the sample size of 60 cases is modest, it is sufficient to evaluate short-term metrics such as surgical time and the learning curve, offering meaningful guidance for surgeons adopting this technology. While long-term functional outcomes and patient satisfaction were not assessed, the study provides a strong foundation for future research and underscores the feasibility and efficiency of integrating imageless RATKA into routine clinical practice.

## Conclusions

This study highlights the feasibility and efficiency of adopting imageless RATKA using VRAS. The findings emphasize that with a structured workflow, consistent training, and surgical team coordination, the learning curve associated with this technology is manageable. The VRAS system allows for precise intraoperative adjustments and personalized implant placement, ensuring optimal soft tissue balance and alignment.

Robotic-assisted technology is not an absolute requirement for performing a technically proficient TKA; however, it provides surgeons with a valuable tool to enhance surgical accuracy, reproducibility, and patient-specific outcomes. By addressing concerns regarding operative time and demonstrating workflow efficiency, this study supports the integration of robotic-assisted systems into routine arthroplasty practice as a means of achieving high-quality results with minimal disruption to established protocols. The potential for improved precision and personalization underscores the role of robotic systems in the future of knee arthroplasty.
